# Antibacterial and Antibiofilm Activity of a Chemically Characterized *Cinnamomum verum* Bark Methanolic Extract Against Multidrug‐Resistant Bacteria: Preliminary Antibiotic–Interaction Screening and Exploratory Molecular Docking

**DOI:** 10.1002/cbdv.71634

**Published:** 2026-08-31

**Authors:** Hayet Edziri, Sarra Elmsehli, Assia kenich, Mabrouk Horchani, Hanen Najjaa, Naima Najah, Hasan Aljohi, Mohammad Nasser Alkhrayef, Roberta Ascrizzi, Guido Flamini, Ibrahim O. Alanazi, Mozaniel Santana de Oliveira

**Affiliations:** ^1^ Laboratory of Transmissible Diseases and Biologically Active Substances, (LR99ES27), Faculty of Pharmacy University of Monastir Monastir Tunisia; ^2^ Laboratory of Organic Chemistry Natural Substances and Analysis (C.O.S.N.A.) Abu Bekr Belkaid University Tlemcen Algeria; ^3^ Laboratory Of Heterocyclic Chemistry, Natural Products and Reactivity, Medicinal Chemistry and Natural Products (LR11ES39), Department of Chemistry, Faculty of Sciences Of Monastir University Of Monastir Monastir Tunisia; ^4^ Institute of Arid Land (IRA) Range Ecology Laboratory Médenine Tunisia; ^5^ Applied Genetics Technology Institute King Abdulaziz City For Science and Technology Riyadh Saudi Arabia; ^6^ Disability Research Institute King Abdulaziz City For Science and Technology Riyadh Saudi Arabia; ^7^ Department of Pharmacy University of Pisa Pisa Italy; ^8^ The Healthy Aging Research Institute King Abdulaziz City For Science and Technology Riyadh Saudi Arabia; ^9^ Laboratory of Pharmacology of Inflammation and Behavior, Institute of Health Sciences, Postgraduate Program in Pharmaceutical Sciences (PPGCF) Federal University of Pará. Belém Pará Brazil

**Keywords:** antibiotic potentiation, antimicrobial resistance, biofilm, *Cinnamomum verum*, molecular docking, phytochemicals

## Abstract

This study characterized the phytochemical composition of a methanolic bark extract of *Cinnamomum verum* and evaluated its antibacterial, bactericidal, biofilm‐biomass‐reducing, and preliminary antibiotic‐potentiating effects against reference and multidrug‐resistant bacterial strains. The extract was characterized by HPLC‐MS; MIC and MBC values were determined by broth microdilution; biofilm biomass was assessed using the crystal‐violet assay; and antibiotic‐extract interactions were screened by disk diffusion. Molecular docking was used as a hypothesis‐generating approach to investigate interactions between identified phytochemicals and GyrB, LasA, and PBP1a. Seventeen compounds were identified, with trans‐cinnamaldehyde as the predominant quantified constituent, followed by quinic acid, rutin, and protocatechuic acid. The extract inhibited all tested bacteria, with MIC and MBC values of 0.625–5 and 2.5–20 mg/mL, respectively, and MBC/MIC ratios consistent with bactericidal activity. Imipenem‐resistant *Acinetobacter baumannii* isolates were the most susceptible. The extract also reduced biofilm biomass in a concentration‐dependent and strain‐dependent manner. Disk‐diffusion screening identified antibiotic‐ and isolate‐dependent increases in inhibition zones, particularly for cefoxitin and fosfomycin against MRSA and for selected combinations against *A. baumannii*; however, some combinations were unchanged or produced smaller zones, and pharmacological synergy was not established. Docking prioritized quercetin, 1,3‐di‐O‐caffeoylquinic acid, and quercetin‐3‐O‐rhamnoside as candidates for subsequent target‐based validation.

## Graphical abstract

The methanolic extract of *Cinnamomum verum* exhibited antibacterial and antibiofilm activities, enhanced the efficacy of conventional antibiotics against multidrug‐resistant bacteria, and showed favorable interactions with key bacterial targets in molecular docking analyses, supporting its potential as a natural antimicrobial adjuvant for combating antibiotic resistance.

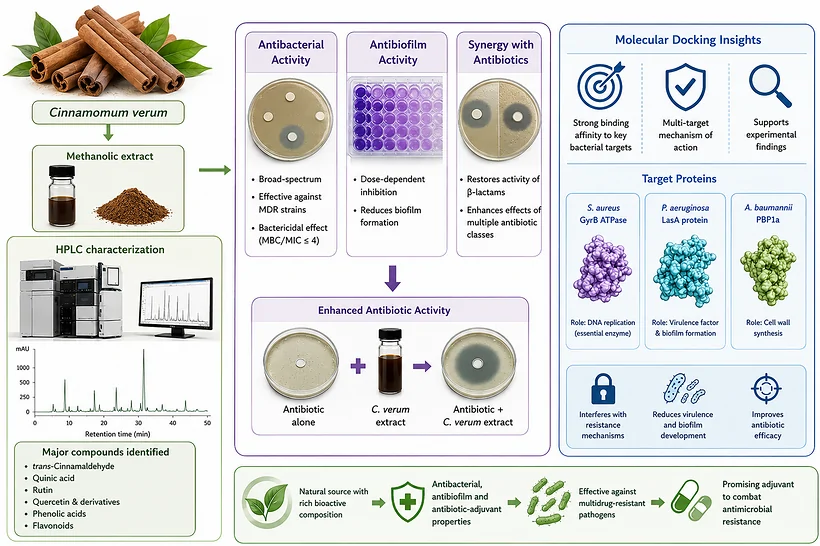

## 1 Introduction

Antimicrobial resistance has become a major challenge in the treatment of bacterial infections, reducing the effectiveness of commonly used antibiotics and increasing the risk of therapeutic failure. Infections caused by methicillin‐resistant *Staphylococcus aureus* (MRSA), multidrug‐resistant *Pseudomonas aeruginosa*, and carbapenem‐resistant *Acinetobacter baumannii* are particularly difficult to manage because these pathogens possess several intrinsic and acquired resistance mechanisms. These mechanisms include reduced membrane permeability, modification of antibiotic targets, production of antibiotic‐inactivating enzymes, active efflux systems, and the acquisition of resistance genes [1, 2, 3].

The clinical persistence of these microorganisms is also associated with their capacity to form biofilms. Within these structured microbial communities, bacterial cells are surrounded by an extracellular matrix that protects them from environmental stress, host immune responses, and antimicrobial agents. Biofilm‐associated bacteria often exhibit lower metabolic activity and increased tolerance to antibiotic treatment compared with planktonic cells [4, 5]. Cell‐to‐cell communication systems, particularly quorum‐sensing‐regulated networks, contribute to bacterial adhesion, motility, extracellular matrix production, virulence‐factor expression, and biofilm maturation [6, 7]. Therefore, compounds capable of reducing bacterial growth, limiting biofilm formation, or improving antibiotic activity are of considerable interest in the search for complementary approaches to control multidrug‐resistant pathogens [8, 9].

Medicinal plants represent an important source of chemically diverse compounds with antimicrobial potential [10, 11, 12]. Phenolic acids, flavonoids, terpenoids, and aromatic aldehydes may interact with bacterial membranes, alter cellular permeability, interfere with energy metabolism, inhibit essential enzymes, and affect processes associated with bacterial virulence [13, 14]. Some plant‐derived compounds may also increase bacterial susceptibility to antibiotics by facilitating antibiotic penetration, affecting membrane integrity, or reducing the activity of bacterial defense mechanisms [15, 16, 17]. Nevertheless, the outcome of plant extract‐antibiotic combinations depend on the composition of the extract, the concentration of each agent, the bacterial strain, its resistance profile, and the antibiotic class evaluated [18, 19].

*Cinnamomum verum* J. Presl, also known by the synonym *Cinnamomum zeylanicum* Blume, belongs to the Lauraceae family and is widely used as a spice, food ingredient, and medicinal plant [20, 21]. The species is traditionally associated with several biological properties, including antimicrobial, anti‐inflammatory, antioxidant, and metabolic effects [22]. Its bark contains a complex mixture of aromatic and phenolic constituents, including cinnamaldehyde, cinnamic acid derivatives, phenolic acids, flavonoids, and other secondary metabolites [23].

Among these constituents, trans‐cinnamaldehyde has received particular attention because of its broad antimicrobial activity. Its hydrophobic structure facilitates interactions with bacterial membranes, potentially altering membrane organization, permeability, and cellular homeostasis [24]. Trans‐cinnamaldehyde has also been investigated as a candidate for overcoming or reducing bacterial resistance, although its activity depends on the microorganism, experimental conditions, and antimicrobial agent used in combination [25]. Other constituents detected in cinnamon extracts, including gallic acid, phenolic acids, rutin, catechin, and related flavonoids may also contribute to biological activity through complementary or multitarget effects [26, 27].

*Cinnamon* essential oils, bark extracts, and purified cinnamaldehyde have previously shown antibacterial activity against gram‐positive and gram‐negative bacteria. However, much of the available literature has focused on essential oils, isolated compounds, reference strains, or the evaluation of a single biological response. Comparatively less information is available on chemically characterized methanolic bark extracts tested against multiple clinically relevant multidrug‐resistant isolates. In particular, the relationship among extract composition, direct bactericidal activity, inhibition of biofilm biomass, and the ability to increase the apparent activity of antibiotics from different pharmacological classes remains insufficiently understood [28, 29, 30].

Another relevant question concerns whether bacterial species with distinct cellular structures and resistance profiles respond similarly to the same cinnamon extract. MRSA, *P. aeruginosa*, and *A. baumannii* differ considerably in terms of membrane organization, efflux capacity, enzymatic resistance mechanisms, biofilm formation, and susceptibility to antimicrobial compounds [31, 32, 33]. A comparative evaluation of these pathogens may therefore provide a clearer understanding of the spectrum and limitations of the antibacterial effects of *C. verum* bark extract.

This study hypothesized that the chemical diversity of the methanolic bark extract of *Cinnamomum verum* could produce species‐dependent antibacterial effects and enhance antibiotic activity. Therefore, we characterized its phytochemical composition by HPLC‐MS and evaluated its inhibitory, bactericidal, antibiofilm, and antibiotic‐potentiating effects against reference and multidrug‐resistant strains of *Staphylococcus aureus*, *Pseudomonas aeruginosa*, and *Acinetobacter baumannii*. Molecular docking was also used as an exploratory approach to investigate potential interactions between the identified compounds and GyrB, LasA, and PBP1a, proteins associated with DNA replication, virulence, and cell‐wall biosynthesis, respectively.

## 2 Materials and Methods

### 2.1 Plant Materials and Preparation of the Extract

*Cinnamomum verum* was purchased at the market of the Medina of Sousse, Tunisia (35°49′40″N 10°38′19″E) in July 2024. Prof. Fethia Skhiri, Botanist at the Monastir Hight Institute of Biotechnology in Tunisia, evaluated the plant's identity. A voucher specimen CV12 was placed at our laboratory at the Faculty of Pharmacy in Monastir. The samples were ground to a mesh size of 1 mm. A 35 g sample of the powdered materials was soaked in 100 mL of methanol for 96 h, then it was filtered and concentrated at reduced pressure using a rotary evaporator. The sample (CV) was subsequently freeze‐dried and it was stored at 4°C for further studies.

### 2.2 Characterization of Extracts

HPLC‐MS analyses were conducted on the methanolic extract of *Cinnamomum verum* using a Shimadzu UFLC XR system (Kyoto, Japan), equipped with an electrospray ionization source (ESI), two LC‐20ADXR pumps, a SIL‐20AXR autosampler, an SCL‐10A system controller, a CTO‐20 AC column oven, and a DGU‐20AS degasser. The phenolic compounds were separated on Bio Wide Pore C_18_ column (150 mm×3 mm, 3 µm) using two mobile phases A and B that consisting of 0.1 aqueous formic acid v/v (phase A) and 0.1% methanolic formic acid v/v (phase B). Following the linear gradient elution: 0–14 min, from 10% to 20 % B; 14–27 min, from 20% to 55% B; 27–37 min, from 55% to 100% B; 37–45 min, 100% B; and 45−50 min 10% B. The MS was operated in negative ion mode with a scanning range from *m/z *35 to *m/z *500 and following the condition: capillary voltage of −3.5 v, a nebulizing gas flow of 1.5 L/min, a dry gas flow rate of 15 L/min, a DL (dissolving line) temperature of 280°C, a block source temperature of 400°C, and a voltage detector of 1.35 V. Data acquisition and processing were performed using Shimadzu LabSolutions software ver.5.42. (Shimadzu, Kyoto, Japan). Quantification and identification of individual compounds were performed by comparing their retention time and mass spectra with those of reference standards and results were expressed as µg per gram of dry weight of the powder extract (µg g^−1^ DW).

### 2.3 Antimicrobial Activity

#### 2.3.1 Bacterial Strain

The micro‐organisms used included three gram‐negative bacteria (*Acinetobacter baumannii* (4), *Pseudomonas aeruginosa* (6), and *Escherichia coli*) and two gram‐positive bacteria (*Enterococcus faecalis* and *Staphylococcus Aureus* SARM (7)). For reference, different strains from the American Type Culture Collection (ATCC) (*Ent. faecalis* (ATCC 29212), *E. coli* (ATCC 25922), *P. aeruginosa* (ATCC 27853), and *Staph. aureus* (ATCC 29213)) were used. All bacterial strains were provided by the Microbiology Laboratory of the University Hospital “Fattouma BOURGUIBA” of Monastir, Tunisia.

#### 2.3.2 Disc Diffusion Technique and Antibiotics Used

The disc diffusion technique described by the Clinical and Laboratory Standards Institute (CLSI, formerly NCCLS) [34], was used to determine the antimicrobial activity of the extracts. This method allows the evaluation of bacterial susceptibility to antimicrobial agents by measuring the inhibition zone formed after diffusion of the compound into the agar medium.

A panel of antibiotics representing different classes was used in this study, including β‐lactams (imipenem, cefoxitin, ticarcillin, ceftazidime, piperacillin, meropenem, and cefotaxime), aminoglycosides (netilmicin and gentamicin), phosphonic acids (Fosfomycin), quinolones (norfloxacin, ciprofloxacin, and ofloxacin), cyclins (tetracycline and tigecycline), sulfonamides (trimethoprim‐sulfamethoxazole), nitrofurans (nitrofurantoin), and polymyxins (colistin). Standard antibiotic discs were used according to their respective concentrations.

For the assay, Mueller‐Hinton agar was poured into sterile Petri dishes (90 mm diameter). A standardized bacterial suspension was inoculated onto the agar surface. Paper discs (6 mm diameter) were impregnated with 5 µL of methanolic extract of *C. zeylanicum* (MEC), while antibiotic discs were applied either alone or in combination with the extract.

After 30 min at room temperature to allow diffusion, the plates were incubated at 37°C for 24 h. The diameter of the inhibition zones was then measured in millimeters. To preliminarily screen for changes in antibiotic activity, 5 µL of MEC was added directly onto the antibiotic discs, and the inhibition zones obtained for the combinations were compared with those produced by the corresponding antibiotics tested alone. The extract alone was also evaluated to determine its intrinsic antimicrobial activity.

#### 2.3.3 Determination of Minimum Inhibitory Concentration (MIC) and Minimum Bactericidal Concentration (MBC)

MICs and MBCs were determined using a microdilution assay as described by Edziri et al., [35]. In the 96‐well plate, 100 µL of nutrient broth were distributed. In the first row of the plate, 200 µL of the methanolic extract stock solution were added. Serial twofold dilutions were subsequently performed. 100 µL of the already prepared bacterial suspension were added to each well and the density adjusted to 0.5 on the McFarland scale. Culture medium alone was used as a negative control and a mixture of medium and bacterial culture as a positive control. The plates were incubated for 18 h at 37°C. The MIC was determined as the lowest concentration at which no growth or turbidity was observed. In order to determine the Minimum Bactericidal Concentration (MBC), 20 µL of each well that showed no visible growth were reinoculated onto Müller‐Hinton agar (MH) plates and incubated at 37°C for 24 h. After incubation, the lowest concentration at which no visible growth was visible was recorded as MBC.

#### 2.3.4 Inhibition of Biofilm Formation

The inhibition of biofilm formation was tested using the modified micro dilution method [36]. Overnight cultures grown in Brain Heart Infusion (BHI) were diluted to 106 CFU/ml in BHI supplemented with 2% glucose (w/v). A 200 µL aliquot was transferred to a 96‐well microtiter plate, and 100 µL of different concentrations of extracts were added. The medium without extract was used as a control. Each assay was repeated three times. After incubation at 37°C for 24 h, the culture supernatant was discarded, and the wells were washed twice with phosphate‐buffered saline (PBS) to remove non adherent cells. The plates were air‐dried, and the cells adhered to the surface were stained with 200 µL of 0.1% crystal violet for 30 min. Then, the crystal violet was removed by washing the plate with water. Crystal violet‐stained biofilm cells were determined at 570 nm with a Multiskan reader (BioRad, Tokyo, Japan).

### 2.4 Molecular Docking Procedure

Molecular docking was performed as an exploratory approach to investigate whether the phytochemicals identified in the methanolic extract of Cinnamomum verum could interact with bacterial proteins associated with processes relevant to the biological effects observed in vitro. The selected targets represent distinct bacterial functions rather than a common signaling pathway. The GyrB ATPase domain of *S. aureus* was selected because it is an essential component of DNA gyrase and plays a central role in bacterial DNA replication [37]. LasA from *Pseudomonas aeruginosa* was included as a zinc‐dependent M23 metalloprotease associated with bacterial virulence and tissue damage; however, LasA is not a quorum‐sensing receptor or a direct component of cell‐to‐cell communication [38]. The penicillin‐binding protein PBP1a from *Acinetobacter baumannii* was selected because of its role in peptidoglycan biosynthesis and its potential relevance to the increased activity observed for some β‐lactam antibiotics in the presence of the extract [39]. Thus, the docking analysis was designed to explore complementary mechanisms potentially associated with direct antibacterial activity, virulence modulation, and cell‐wall biosynthesis.

The crystal structures of the *S. aureus* GyrB ATPase domain in complex with a small‐molecule inhibitor (PDB ID: 3U2D), the LasA virulence factor from *P. aeruginosa* (PDB ID: 3IT7), and *A. baumannii* PBP1a in complex with penicillin G (PDB ID: 3UDI) were obtained from the RCSB Protein Data Bank. Molecular docking simulations were carried out using AutoDock 4.2 [40]. Before docking, the co‐crystallized ligands and water molecules were removed from the protein structures. Polar hydrogen atoms were added, Gasteiger partial charges were assigned, and the receptor structures were converted to PDBQT format using AutoDockTools.

The three‐dimensional structures of the phytochemicals identified by HPLC‐MS were generated and geometrically optimized using ACD/3D Viewer. The ligands were subsequently prepared in AutoDockTools by assigning rotatable bonds and Gasteiger charges and converting the structures to PDBQT format. Grid maps were generated using AutoGrid, with the grid boxes centered on the binding regions occupied by the respective co‐crystallized ligands and adjusted to encompass the corresponding active‐site residues.

The docking protocol was evaluated by redocking the native ligand of each protein into its original binding site and comparing the predicted pose with the experimentally determined crystallographic orientation. The agreement between the redocked and crystallographic poses was assessed by superposition and root‐mean‐square deviation analysis. The same docking parameters were subsequently applied to the phytochemicals identified in the extract. The most representative poses were selected by considering the predicted binding energy, ligand orientation within the binding pocket, and interactions with relevant active‐site residues.

Protein‐ligand interactions were visualized and analyzed using Discovery Studio 2017 R2 and PyMOL 0.99rc6. Because molecular docking provides predicted binding modes and relative affinity estimates, the results were interpreted as hypothesis‐generating evidence and not as direct confirmation of target inhibition or mechanism of action. Experimental enzymatic, biochemical, or molecular assays would be required to validate the predicted interactions.

### 2.5 Statistical Analysis

All experiments were repeated three times and results are averaged ± SD (standard deviation). One‐way ANOVA and the Tukey test were used to assess the data using IBM SPSS Statistics 20. Statistical significance was defined as a p value of less than 0.05.

## 3 Results and Discussion

### 3.1 Determination of the Composition of the Methanolic Cinnamon Bark Extract by HPLC

**FIGURE 1:**
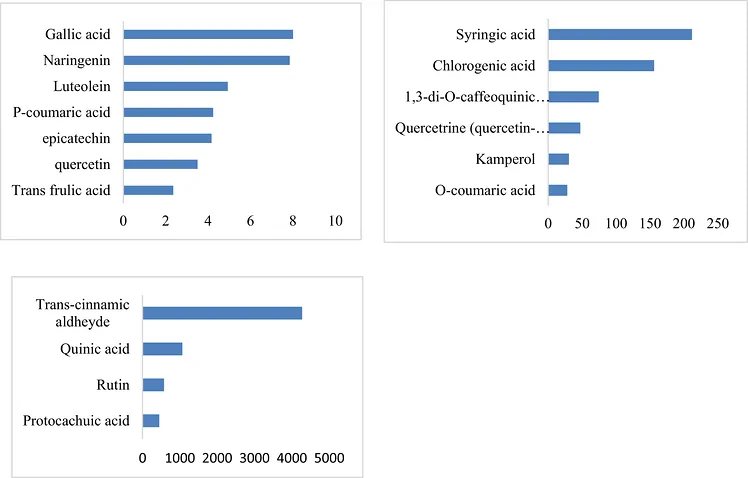
HPLC analysis of phenolic composition of *C. verum* bark (ppm).

HPLC‐MS analysis revealed a chemically diverse profile comprising 17 compounds. Trans‐cinnamaldehyde was the predominant quantified constituent (4275.934 µg g^−1^ DW), followed by quinic acid (1068.742 µg g^−1^ DW), rutin (577.950 µg g^−1^ DW), and protocatechuic acid (450.175 µg g^−1^ DW). The predominance of *trans*‐cinnamaldehyde is particularly relevant to the antibacterial findings because this aromatic aldehyde has been associated with membrane perturbation, altered cellular permeability, and disruption of bacterial homeostasis [41, 42, 43]. The presence of phenolic acids and flavonoids may also contribute to the overall activity of the extract through complementary interactions with bacterial membranes, enzymes, and other cellular components [44, 45]. Nevertheless, the biological contribution of each constituent cannot be established solely from its concentration in the extract. The concentrations of all identified constituents are presented in Table 1 and the corresponding HPLC profile is shown in Figure 1.

**TABLE 1:** Contents of phenolic compounds (µg g^−1^ of DW) determined by HPLC analysis of *C. verum* methanolic extract.

N	Compounds	Retention time	Concentration (ppm)	m/z
**1**	Quinic acid	1.585	1068.742	191
**2**	1,3‐di‐O‐caffeoquinic acid	1.619	74.678	515
**3**	Gallic acid	1.625	8.01	169
**4**	Protocachuic acid	4.602	450.175	153
**5**	Chlorogenic acid	8.635	156.248	353
**6**	Syringic acid	11.873	212.158	197
**7**	Epicatechin	12.716	4.167	289
**8**	P‐coumaric acid	16.325	4.238	163
**9**	*Trans*‐frulic acid	19.53	2.359	193
**10**	Rutin	21.666	577.95	609
**11**	O‐coumaric acid	22.964	28.204	163
**12**	Quercetrine (quercetin‐ 3‐o‐rhamonoside)	24.179	47.454	447
**13**	Quercetin	29.099	3.503	301
**14**	*Trans*‐cinnamic aldheyde	29.19	4275.934	147
**15**	Kaempferol	29.212	30.752	285
**16**	Naringenin	31.407	7.854	271
**17**	Luteolein	32.288	4.929	285

Rutin, a glycosylated flavonoid (577.950 µg g^−1^), is for its robust antioxidant effect, as well as its vaso‐protective, antihypertensive, and anti‐inflammatory actions. The presence of phenolic acids, including protocatechuic (450.175 µg g^−1^), chlorogenic (156.248 µg g^−1^), syringic (212.158 µg g^−1^), and 1.3‐di‐O‐caffeoylquinic (74.678 µg g^−1^), the interactions among the constituents should not be described as synergistic without testing the isolated compounds and their combinations. These compounds have been identified as effective free radical sequestering agents, capable of inhibiting oxidative enzymes and modulating cell signaling mechanisms involved in inflammatory and degenerative processes [46, 47, 48, 49].

Aglycone flavonoids, such as quercetin (3.503 µg g^−1^), kaempferol (30.752 µg g^−1^), naringenin (7.854 µg g^−1^), and luteolin (4.929 µg g^−1^), although present in lower concentrations, have been shown to possess significant bioactivity and exert pleiotropic effects, including modulation of apoptotic pathways, inhibition of uncontrolled cell proliferation, and cardiovascular protection [50, 51]. Quercitrin (47.454 µg g^−1^), the glycosylated form of quercetin, exhibits superior solubility and bioavailability, thereby promoting its absorption and therapeutic potential in vivo. Epicatechin (4.167 µg g^−1^), a catechin has been linked to enhanced endothelial function, reduced blood pressure, and neuroprotection [52].

The presence of compounds such as *p*‐ and *o*‐coumaric acids, ferulic acid, and gallic acid, even in lower concentrations, complements the antioxidant and anti‐inflammatory profile of the extract, giving it a broad and diverse functional spectrum. These compounds play an important role in preventing cellular oxidative stress and are associated with reducing the risk of chronic non‐communicable diseases such as diabetes, cancer, and cardiovascular diseases [53]. The chemical complexity extract may also act to modulate the intestinal microbiota, protect against exacerbated oxidative processes, and attenuate deregulated immune responses. Consequently, the findings substantiate the long‐standing utilization of *C. verum* and underscore the imperative for further exploration, encompassing in vitro and in vivo studies and clinical trials, to elucidate the mechanisms of action of its primary constituents and to investigate their potential applications in pharmaceutical, nutraceutical, and cosmeceutical formulations [54, 55, 56].

#### 3.1.1 Antimicrobial Activity: Determination of MIC, MBC, and MBC/MIC Ratio

Table 2 summarizes the antimicrobial activity of the methanolic extract of *C. verum* against gram‐positive and gram‐negative bacteria, including several multidrug‐resistant isolates. MIC values ranged from 0.625 to 5 mg/mL, whereas MBC values ranged from 2.5 to 20 mg/mL. After correction of the MBC/MIC ratios, all values were either 2 or 4. According to the interpretative criterion adopted in this study, an MBC/MIC ratio of ≤4 indicates a bactericidal effect [57]. However, this classification reflects the relationship between the inhibitory and bactericidal concentrations and does not provide information about the rate of bacterial killing, which would require time‐kill assays.

**TABLE 2:** Antimicrobial activity of methanolic extract of *C. verum*.

Strains		MIC (mg/mL)	MBC (mg/mL)	MBC/MIC
Gram positive bacteria	*Enterococcus faecalis* ATCC 29212	1.25	2,5	2
	*Staphylococcus aureus* ATCC 29213	2.5	10	4
	*Staphylococcus aureus* MRSA 2953	2.5	10	4
	*Staphylococcus aureus* MRSA HFB 4945	2.5	10	4
	*Staphylococcus aureus* MRSA HFB 8064	5	20	4
	*Staphylococcus aureus* MRSA A 4620	5	20	4
	*Staphylococcus aureus* MRSA A 3443	5	20	4
	*Staphylococcus aureus* MRSA A 6960	5	20	4
	*Staphylococcus aureus* MRSA A 2861	5	20	4
Gram negative bacteria	*Escherichia coli* ATCC 25922	5	10	2
	*Pseudomonas aeruginosa* ATCC 27853	2,5	10	4
	*Pseudomonas aeruginosa* IMP/R	5	10	2
	*Pseudomonas aeruginosa* IMP/R	5	10	2
	*Pseudomonas aeruginosa* RC A 861	5	20	4
	*Pseudomonas aeruginosa* RC A 443	5	20	4
	*Pseudomonas aeruginosa* RC 862	5	10	2
	*Pseudomonas aeruginosa* RC A 942	5	10	2
	*Acinetobacter baumannii* IMP/R A722	1.25	5	4
	*Acinetobacter baumannii* IMP/R A2172	1.25	5	4
	*Acinetobacter baumannii* IMP/R A 2553	0.625	2.5	4
	*Acinetobacter baumannii* IMP/R B2049	0.625	2.5	4

Among the gram‐positive bacteria, *Enterococcus faecalis* ATCC 29212 was the most susceptible strain, with an MIC of 1.25 mg/mL and an MBC of 2.5 mg/mL. The reference *S. aureus* strain and the MRSA isolates showed MIC values ranging from 2.5 to 5 mg/mL and MBC values ranging from 10 to 20 mg/mL. Some MRSA isolates showed the same MIC as the reference *S. aureus* strain, whereas others required a two‐fold higher concentration. These findings indicate that methicillin resistance was not uniformly associated with reduced susceptibility to the extract, although differences among the MRSA isolates suggest that their individual resistance profiles or physiological characteristics may have influenced the response.

Susceptibility among the gram‐negative bacteria was more variable. *E. coli* ATCC 25922 showed an MIC of 5 mg/mL and an MBC of 10 mg/mL. The *P. aeruginosa* strains exhibited MIC values between 2.5 and 5 mg/mL and MBC values between 10 and 20 mg/mL. After correction, their MBC/MIC ratios ranged from 2 to 4, supporting a bactericidal classification according to the criterion used in this study. Nevertheless, the relatively high concentrations required to inhibit most *P. aeruginosa* isolates are consistent with the reduced susceptibility commonly associated with this species.

Among all tested microorganisms, the multidrug‐resistant *A. baumannii* isolates showed the lowest MIC values, ranging from 0.625 to 1.25 mg/mL, and MBC values ranging from 2.5 to 5 mg/mL. Isolates A2553 and B2049 were particularly susceptible, with MIC and MBC values of 0.625 and 2.5 mg/mL, respectively. These results indicate greater susceptibility of the tested *A. baumannii* isolates to the extract under the experimental conditions used. However, this finding should be interpreted as a strain‐dependent response and not as evidence that the extract is generally more active against gram‐negative than gram‐positive bacteria.

The antibacterial activity of the *C. verum* methanolic extract is likely related to the combined contribution of its chemically diverse constituents. Phenolic acids, flavonoids, and aromatic aldehydes have been associated with alterations in bacterial membrane integrity, permeability, enzyme activity, and cellular homeostasis [58, 59, 60]. Nevertheless, the contribution of each compound cannot be established from the activity of the crude extract alone, and the interactions among the constituents should not be described as synergistic without testing the isolated compounds and their combinations.

*Trans*‐cinnamaldehyde, the most abundant compound detected in the extract, may have made an important contribution to the observed activity. Its aromatic aldehyde group and hydrophobic structure favor interactions with lipid components of bacterial membranes. These interactions may alter membrane organization and permeability, promote the loss of intracellular components, and interfere with membrane‐associated cellular processes [61, 62]. Such effects may partly explain the bactericidal activity observed against both gram‐positive and gram‐negative strains.

Other constituents, including protocatechuic acid, rutin, quercitrin, epicatechin, and quercetin may also contribute through complementary mechanisms. Depending on their physicochemical properties, phenolic compounds and flavonoids may interact with bacterial membranes, proteins, metabolic enzymes, or intracellular targets [63, 64, 65]. However, their actual contribution depends not only on their intrinsic biological activity but also on their concentration, stability, solubility, and accessibility to bacterial targets within the crude extract.

Overall, the MIC and MBC results demonstrate that the methanolic extract of *C. verum* exerted bactericidal activity against the evaluated reference and multidrug‐resistant strains, with the greatest susceptibility observed among the tested *A. baumannii* isolates. However, the active concentrations remained within the mg/mL range. Further studies involving extract fractionation, evaluation of isolated constituents, time‐kill analysis, cytotoxicity testing, and determination of selectivity indices are therefore needed to clarify the biological relevance and potential applicability of these findings.

### 3.2 Antibiofilm Activity of Cinnamon Methanolic Extract

**FIGURE 2:**
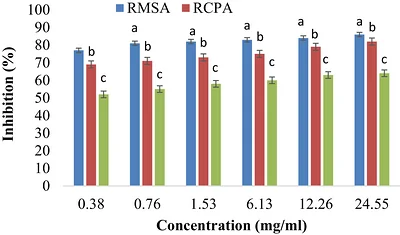
Effect of the methanolic extract of Cinnamomum verum on biofilm biomass produced by multidrug‐resistant bacterial strains. MRSA: methicillin‐resistant Staphylococcus aureus; RCPA: ceftazidime‐resistant Pseudomonas aeruginosa; RIAB: imipenem‐resistant *Acinetobacter baumannii*. Results are expressed as mean ± standard deviation (*n* = 3). Different letters indicate significant differences among extract concentrations for the same bacterial strain according to Tukey's test (*p* < 0.05).

Biofilm development involves a sequence of events that includes initial adhesion, cellular proliferation, extracellular matrix production, and biofilm maturation. This organization improves bacterial survival under unfavorable conditions and limits the penetration and effectiveness of antimicrobial agents and other toxic substances [66, 67]. Figure 2 shows the effect of the methanolic extract of *C. verum* on biofilm biomass produced by multidrug‐resistant MRSA, ceftazidime‐resistant P. aeruginosa, and imipenem‐resistant *A. baumannii*. The extract reduced the amount of attached biomass in a concentration‐dependent manner, with significant differences among the tested concentrations (*p* < 0.05).

The magnitude of biofilm inhibition varied among the bacterial strains, suggesting that susceptibility to the extract was influenced by species‐specific characteristics [68]. Differences in cell‐envelope structure, surface adhesion, extracellular matrix composition, and biofilm‐regulatory pathways may have contributed to the responses observed. However, the crystal‐violet assay measures the total biomass attached to the microplate surface and does not distinguish viable bacterial cells from extracellular matrix components or nonviable cells [69].

This limitation is particularly relevant because some of the concentrations tested were equal to or higher than the MIC values determined for the corresponding strains. Therefore, at the highest concentrations, the reduction in crystal‐violet staining may have resulted, at least partly, from inhibition of planktonic growth or bacterial killing rather than from a specific effect on biofilm formation [70, 71]. The responses observed at sub‐inhibitory concentrations provide more relevant preliminary evidence that the extract may affect adhesion, matrix production, or early stages of biofilm development. Nevertheless, additional assays normalizing biofilm formation to bacterial growth or viability would be required to distinguish a specific antibiofilm effect from the direct antibacterial activity of the extract [72, 73].

The high concentration of *trans*‐cinnamaldehyde in the extract may have contributed to the reduction in biofilm biomass. Previous studies have associated this compound with decreased bacterial motility, reduced exopolysaccharide production, and alterations in the formation and function of flagella [74, 75]. These effects could impair initial surface colonization and subsequent biofilm maturation. Other phenolic compounds and flavonoids detected in the extract may also contribute through complementary effects on bacterial membranes, adhesion‐related proteins, or regulatory pathways. However, the contribution of individual constituents was not determined in the present study.

Quorum sensing is a cell‐to‐cell communication mechanism based on the production, release, and detection of signaling molecules. These signaling networks regulate several bacterial behaviors, including motility, production of virulence factors, extracellular matrix synthesis, and biofilm development [76]. Previous investigations have reported that *trans*‐cinnamaldehyde and cinnamon bark preparations can affect quorum‐sensing‐regulated responses in *P. aeruginosa*, including the production of signaling molecules and biofilm‐associated phenotypes [77]. Nevertheless, quorum‐sensing molecules and the expression of quorum‐sensing‐related genes were not directly measured in the present study. Therefore, the current results demonstrate a reduction in biofilm biomass but do not confirm that this effect occurred through quorum‐sensing inhibition. Further studies involving bacterial viability, signaling‐molecule quantification, microscopy, and analysis of biofilm‐ and quorum‐sensing‐related gene expression are needed to clarify the underlying mechanisms.

### 3.3 Preliminary Assessment of Antibiotic‐extract Interactions by Disk Diffusion

Tables 3 and 4 summarize the changes in inhibition‐zone diameters observed when the methanolic extract of *C. verum* (MEC) was combined with antibiotics against imipenem‐resistant *A. baumannii* and methicillin‐resistant *S. aureus*. The disk‐diffusion assay was used as a preliminary screening method to identify combinations associated with increased antibacterial activity. Because inhibition‐zone diameters are influenced by the diffusion properties of both the antibiotic and the constituents of the crude extract, this method does not provide quantitative confirmation of pharmacological synergy. Therefore, the results were interpreted as preliminary evidence of antibiotic potentiation only when the inhibition zone produced by the combination exceeded those produced by both the antibiotic and MEC tested separately.

**TABLE 3:** Preliminary disk‐diffusion assessment of interactions between the methanolic extract of *Cinnamomum verum* and antibiotics against imipenem‐resistant *Acinetobacter baumannii*.

Antibiotic disc		Inhibition zone (mm): Only ATB / ATB + ME of *C. zeylanicum*							
		*Acinetobacter baumannii* imipenem resistant.							
		ABIR A 722		ABIR A 2172		ABIR A 2553		ABIR B 2049	
	Inhibition zone ATB (mm)	ATB only	ATB +extract	ATB Only	ATB +extract	ATB only	ATB +extract	ATB only	ATB + extract
β‐lactam antibiotics	Imipenem (10 µg)	13	14	10	13	10	14	9	21
	Cefoxitin (30 µg)	0	14	0	15	0	13	0	18
	Ticarcillin (75 µg)	0	14	0	17	0	13	0	17
	Ceftazidime (10 µg)	0	15	0	18	0	13	0	13
	Piperacillin (30 µg)	0	14	0	17	0	15	0	14
	Meropenem (10 µg)	0	14	0	15	0	13	0	15
	Cefotaxime (10 µg)	0	14	0	15	0	13	0	12
Aminoglycosides	Netilmicin (10 µg)	0	15	0	21	26	30	0	12
	Gentamicin (10 µg)	0	13	0	0	11	13	0	12
Phosphonic acids	Fosfomycin (200 µg)	21	24	15	19	40	42	14	17
Quinolones	Norfloxacin (10 µg)	0	14	0	22	27	30	0	0
	Ciprofloxacin (5 µg)	0	15	0	26	30	33	0	11
	Ofloxacin (5 µg)	0	14	0	23	24	28	0	11
Cyclins	Tetracycline (30 µg)	0	14	0	17	0	13	0	11
	Tigecyclin (15 µg)	14	15	13	15	26	30	0	15
Sulfonamides	Trimethoprim sulfanetoxazole (25 µg)	0	14	0	15	26	28	0	11
Nitrofurans	Nitrofurantoin (100 µg)	0	14	0	0	30	28	0	0
Polymyxin	Colistin (50 µg)	21	22	15	17	20	22	15	16
ME of only *C. verum* (60 µl)		14		13		14		11	

Values represent inhibition‐zone diameters expressed in millimeters.

Abbreviations: MEC, methanolic extract of Cinnamomum verum; ATB, antibiotic. The disk‐diffusion assay was used as a preliminary screening method and does not independently confirm pharmacological synergy.

**TABLE 4:** Preliminary disk‐diffusion assessment of interactions between the methanolic extract of *Cinnamomum verum* and antibiotics against methicillin‐resistant *Staphylococcus aureus*.

Antibiotics discs		Inhibition zone (mm) : Only ATB / ATB + ME of *C. zeylanicum*									
		*Staphylococcus aureus* Methicillin‐resistant									
		MRSA A2953		MRSA A4620		MRSA A3443		MRSA A2861		MRSA A6960	
	Inhibition zone ATB (mm)	ATB seul	ATB + extrait	ATB seul	ATB + extrait	ATB seul	ATB + extrait	ATB seul	ATB + extrait	ATB seul	ATB + extrait
β‐lactam antibiotics	Cefoxilin (30 µg)	23	27	20	29	18	25	20	27	17	20
	Penicillin G (10 µg)	0	15	0	14	0	16	0	15	0	18
Macrolides and similers	Clindamycin (2 µg)	34	37	23	31	22	26	17	24	22	26
	Erythromycin (15 µg)	32	36	27	40	15	23	24	26	30	32
Aminoglycosides	Kanamycin (15 µg)	23	30	29	24	23	25	23	27	24	28
Fusidic acid	Fusidic acid (10 µg)	34	40	34	34	14	22	26	30	31	34
Quinolones	Norfloxacin (10 µg)	32	36	32	34	24	20	30	32	28	30
	Ofloxacin (5 µg)	28	34	31	36	30	35	33	37	29	32
Phosphonic acids	Fosfomycin (200 µg)	29	35	40	48	33	45	40	49	42	50
Cyclins	Minocyclin	27	33	30	36	22	26	25	27	22	25
Sulfonamides	Trimethoprim sulfanetoxazole (25 µg)	33	27	30	40	20	25	26	29	27	30
Synergistins	Quinupristin –Dalfopristin (15 µg)	28	34	32	36	32	34	19	28	32	32
Oxozolidinones	Linezolide (10 µg)	30	34	34	36	30	33	22	24	24	26
Rifampicin	Rifampicin (5 µg)	29	33	33	37	17	23	23	28	18	24
ME of *C. Verum* only (60 µL)		15		14		16		15		18	

Values represent inhibition‐zone diameters expressed in millimeters.

Abbreviations: MEC, methanolic extract of Cinnamomum verum; ATB, antibiotic. The disk‐diffusion assay was used as a preliminary screening method and does not independently confirm pharmacological synergy.

Several β‐lactam antibiotics that produced no measurable inhibition zones when tested alone yielded detectable zones after the addition of MEC. However, the magnitude of this response varied considerably among the isolates. Against ABIR A722, for example, the combinations containing cefoxitin, ticarcillin, piperacillin, meropenem, cefotaxime, and tetracycline produced inhibition zones of 14 mm, which was identical to the zone produced by MEC alone. In these cases, the observed activity can primarily be attributed to the intrinsic antibacterial effect of the extract, and restoration of antibiotic susceptibility cannot be inferred.

More pronounced increases were observed against ABIR B2049. The inhibition zone of imipenem increased from 9 mm when tested alone to 21 mm in combination with MEC, exceeding the 11‐mm zone produced by the extract alone. Cefoxitin, ticarcillin, and meropenem, which were inactive when tested individually, produced inhibition zones of 18, 17, and 15 mm, respectively, when combined with MEC. These values were also greater than that obtained with the extract alone, indicating potentially relevant antibiotic–extract interactions that warrant quantitative confirmation.

The response of ABIR A2172 also suggested preliminary potentiation for selected β‐lactams. Ticarcillin and piperacillin produced inhibition zones of 17 mm in combination with MEC, whereas ceftazidime produced a zone of 18 mm. These antibiotics showed no detectable activity when tested alone, while MEC alone produced a 13‐mm inhibition zone. Nevertheless, the difference between the combination and the extract alone was relatively modest for some antibiotics and should not be interpreted as confirmed synergy.

Selected aminoglycosides and quinolones also showed isolate‐dependent increases. Against ABIR A2172, netilmicin, ciprofloxacin, and ofloxacin were inactive when tested alone but produced inhibition zones of 21, 26, and 23 mm, respectively, in the presence of MEC. These values were substantially greater than the 13‐mm zone produced by MEC alone. Against ABIR A2553, netilmicin, ciprofloxacin, and ofloxacin showed increases of approximately 3–4 mm relative to the corresponding antibiotics alone, suggesting a more modest potentiating effect.

Fosfomycin showed small but consistent increases across the four *A. baumannii* isolates. The inhibition zones increased from 21 to 24 mm for ABIR A722, from 15 to 19 mm for ABIR A2172, from 40 to 42 mm for ABIR A2553, and from 14 to 17 mm for ABIR B2049. Although these differences indicate increased activity in the presence of MEC, the relatively limited changes require confirmation by quantitative methods before a synergistic interaction can be assigned.

Colistin showed increases of only 1‐2 mm when combined with MEC, suggesting limited potentiation under the conditions used. Other combinations did not improve antibacterial activity. Gentamicin remained inactive against ABIR A2172, norfloxacin remained inactive against ABIR B2049, and nitrofurantoin showed no improvement against ABIR A2172 and ABIR B2049. Against ABIR A2553, the inhibition zone of nitrofurantoin decreased from 30 to 28 mm after the addition of MEC. These findings demonstrate that the influence of the extract depended on both the antibiotic and the bacterial isolate.

The antibiotic‐extract interactions against MRSA were also dependent on the antibiotic and bacterial isolate. Cefoxitin produced larger inhibition zones in combination with MEC across all five MRSA isolates. The increases relative to cefoxitin alone ranged from 3 to 9 mm, and the zones produced by the combinations were greater than those obtained with MEC alone. These findings indicate preliminary potentiation of cefoxitin activity, although quantitative assays are required to determine the nature of the interaction.

In contrast, the apparent activity of penicillin G in the combined treatment was entirely attributable to MEC. Penicillin G produced no measurable inhibition zones when tested alone, whereas the inhibition zones of the combinations were 15, 14, 16, 15, and 18 mm. These values were identical to those produced by MEC alone against the respective MRSA isolates. Therefore, the results do not demonstrate restoration of penicillin G activity or potentiation of this antibiotic.

Clindamycin and erythromycin generally showed larger inhibition zones in the presence of MEC. The most pronounced increase was observed for erythromycin against MRSA A4620, for which the zone increased from 27 to 40 mm. Clindamycin increased from 23 to 31 mm against the same isolate and from 17 to 24 mm against MRSA A2861. These findings indicate potentially relevant interactions, but their statistical and pharmacological significance cannot be determined from inhibition‐zone diameters alone.

Fosfomycin showed the most consistent increases among the antibiotics tested against MRSA. Inhibition‐zone diameters increased by 6–12 mm across the five isolates, and all combinations produced zones substantially greater than those observed with MEC alone. Ofloxacin, minocycline, linezolid, and rifampicin also generally showed larger inhibition zones when combined with the extract, although the magnitude of the increases varied among isolates.

Not all combinations resulted in improved activity. The inhibition zone of kanamycin against MRSA A4620 decreased from 29 to 24 mm, norfloxacin against MRSA A3443 decreased from 24 to 20 mm, and trimethoprim‐sulfamethoxazole against MRSA A2953 decreased from 33 to 27 mm. Quinupristin‐dalfopristin showed no change against MRSA A6960. These results reinforce that the effects of MEC were not uniformly beneficial and that each antibiotic–isolate combination should be evaluated individually.

Overall, the disk‐diffusion results identified several combinations that merit further investigation, particularly imipenem, selected β‐lactams, netilmicin, and quinolones against some A. baumannii isolates, as well as cefoxitin, erythromycin, clindamycin, and phosphomycin against MRSA. However, disk diffusion cannot distinguish synergistic, additive, indifferent, or antagonistic interactions. Checkerboard microdilution with determination of the fractional inhibitory concentration index, followed by time‐kill assays for the most promising combinations, is required to quantitatively confirm the nature of these interactions. Until such assays are performed, the results should be described as preliminary antibiotic potentiation rather than confirmed synergy.

#### 3.3.1 Antibiotic‐Extract Interactions Against Methicillin‐resistant *Staphylococcus aureus*

The influence of the methanolic extract of *C. verum* on antibiotic activity against MRSA varied according to the antibiotic and bacterial isolate. Several combinations produced larger inhibition zones than the corresponding antibiotics tested alone, particularly those involving cefoxitin, clindamycin, erythromycin, ofloxacin, fosfomycin, minocycline, linezolid, and rifampicin. However, because these interactions were evaluated by disk diffusion, the observed increases should be interpreted as preliminary evidence of antibiotic potentiation rather than quantitative confirmation of synergy.

Penicillin G showed no measurable activity when tested alone against any of the MRSA isolates. When combined with the extract, inhibition zones ranging from 14 to 18 mm were observed. Nevertheless, these values were identical to those produced by the extract alone against the corresponding isolates. Therefore, the inhibition observed for the combined disks was attributable to the intrinsic antibacterial activity of the extract and does not demonstrate restoration of penicillin G susceptibility.

Among the β‐lactams, cefoxitin showed a more consistent pattern of potentiation. Its inhibition zones increased in the presence of the extract across all MRSA isolates, and the combined treatments produced larger zones than either cefoxitin or the extract tested separately. The magnitude of this increase differed among isolates, indicating that the interaction was dependent on the bacterial resistance profile. Similar isolate‐dependent responses were observed for clindamycin and erythromycin, with the largest increases recorded against MRSA A4620 and MRSA A3443.

Fosfomycin showed the most consistent enhancement among the antibiotics evaluated against MRSA. The addition of the extract increased inhibition‐zone diameters across all five isolates, with the combined treatments producing zones considerably larger than those obtained with the extract alone. Ofloxacin, minocycline, linezolid, and rifampicin also generally showed moderate increases in activity. These combinations may therefore be considered candidates for further quantitative investigation.

Conversely, the extract did not improve the activity of all antibiotics. The inhibition zone of kanamycin decreased against MRSA A4620, norfloxacin activity decreased against MRSA A3443, and trimethoprim–sulfamethoxazole showed a reduced inhibition zone against MRSA A2953. No improvement was observed for quinupristin‐dalfopristin against MRSA A6960. These findings demonstrate that the effects of the extract were not uniformly beneficial and that antagonistic or indifferent interactions cannot be excluded for some combinations.

The antibiotic‐potentiating effects observed for selected combinations may be associated with the chemically diverse constituents of the extract. Previous studies have suggested that plant‐derived compounds can disrupt membrane organization, alter membrane permeability, promote leakage of intracellular components, modify surface charge, and induce protein denaturation [78, 79]. Such alterations could facilitate antibiotic access to intracellular or membrane‐associated targets. However, these mechanisms were not directly investigated in the present study and should therefore be regarded as possible explanations rather than experimentally demonstrated effects.

The methanolic bark extract of *C. verum* exhibited strain‐dependent antibacterial activity against the tested reference and multidrug‐resistant bacteria. Devara et al. similarly demonstrated that methanolic and ethanolic cinnamon bark extracts inhibited clinical isolates of *S. aureus*, coagulase‐negative *staphylococci*, *E. coli*, *Klebsiella pneumoniae, Proteus mirabilis, and P. aeruginosa*. In that study, *S. aureus* showed the largest inhibition zones, whereas the methanolic extract presented an MIC of 12.5 mg/mL and MBC values ranging from 12.5 to 50 mg/mL [80]. Although the greater susceptibility of *S. aureus* reported by these authors differs from the lower MIC values observed for the *A. baumannii* isolates than for most MRSA isolates in the present study, this difference is not necessarily contradictory. Al‐Garadi et al. reported MIC values of 3.26–7.81 mg/mL for gram‐negative bacteria and values ranging from 6.51 to 62.50 mg/mL for gram‐positive bacteria exposed to ethanolic or aqueous *C. verum* bark extracts [81]. Collectively, these findings indicate that susceptibility to cinnamon extracts cannot be predicted solely from Gram classification and should be interpreted according to the bacterial species, strain, resistance phenotype, extract composition, and experimental conditions.

Considerable variation in antimicrobial potency has been reported among cinnamon preparations. Ahuokpeme et al. [82] found an MIC of 25 mg/mL for both aqueous and ethanolic *C. zeylanicum* bark extracts against *S. aureus*, *P. aeruginosa*, *E. coli*, *Bacillus* sp., and *Candida albicans*. However, the ethanolic extract required 200 mg/mL to produce bactericidal effects against *S. aureus*, *P. aeruginosa*, and *E. coli*, whereas the aqueous extract showed no bactericidal activity at the evaluated concentrations. Waty et al. [83] also reported a concentration‐dependent response for an ethanolic *C. burmannii* bark extract, with inhibition zones of 6.78, 9.00, and 11.68 mm at concentrations of 6.25%, 12.5%, and 25%, respectively, against bacteria isolated from dental plaque. In comparison, tetracycline produced an inhibition zone of 28.3 mm. These differences emphasize that crude‐extract percentages and inhibition zones obtained by agar‐diffusion assays cannot be directly compared with MIC and MBC values generated by dilution methods. They also demonstrate the influence of cinnamon species, solvent polarity, extraction conditions, phytochemical yield, inoculum, culture medium, and bacterial origin.

The effects on biofilm biomass observed in the present investigation are consistent with previous evidence that cinnamon extracts can affect sessile bacterial communities. Panjaitan et al. demonstrated that an ethanolic *C. burmannii* extract significantly reduced biofilms of *Porphyromonas gingivalis* and *Aggregatibacter actinomycetemcomitans* at concentrations ranging from 2.5% to 15%. Even the lowest concentration significantly reduced biofilm biomass, while 7.5% and 2.5% were the most effective concentrations against *P. gingivalis* and *A. actinomycetemcomitans*, respectively [84]. Although these periodontal pathogens differ from the multidrug‐resistant bacteria evaluated in the present study, both investigations support the hypothesis that cinnamon‐derived phytochemicals may interfere with biofilm establishment or maintenance. Nevertheless, differences in cinnamon species, microbial targets, concentrations, and exposure conditions permit only a qualitative comparison between the studies.

The antimicrobial properties of cinnamon have been associated mainly with cinnamaldehyde and other phenylpropanoids and phenolic compounds capable of disturbing membrane integrity, permeability, enzyme activity, energy metabolism, motility, and biofilm‐related processes [85]. Membrane perturbation represents a biologically plausible explanation for the increased activity of selected antibiotics against MRSA when combined with the methanolic extract in the present study, because increased permeability or loss of membrane homeostasis may facilitate antibiotic access to intracellular or membrane‐associated targets. However, this interpretation remains a mechanistic hypothesis because membrane permeability, efflux activity, and intracellular antibiotic accumulation were not directly evaluated.

Overall, the increased inhibition zones observed for selected antibiotics combined with the *C. verum* extract indicate preliminary antibiotic potentiation against MRSA. Importantly, none of the comparative studies discussed above quantitatively evaluated cinnamon extract–antibiotic combinations using fractional inhibitory concentration indices or time‐kill kinetics. Therefore, these studies support the biological plausibility of the observed interaction but do not independently confirm synergy. Moreover, changes in disk‐diffusion inhibition zones may be influenced by the diffusion properties of both the extract and the antibiotic and cannot distinguish synergistic, additive, indifferent, or antagonistic interactions. Checkerboard microdilution followed by time‐kill assays should therefore be conducted to classify these interactions and determine whether potentiation is maintained over time. Until quantitative confirmation is available, the present findings should be described as preliminary antibiotic potentiation rather than confirmed synergistic activity.

### 3.4 Molecular Docking Studies

**FIGURE 3:**
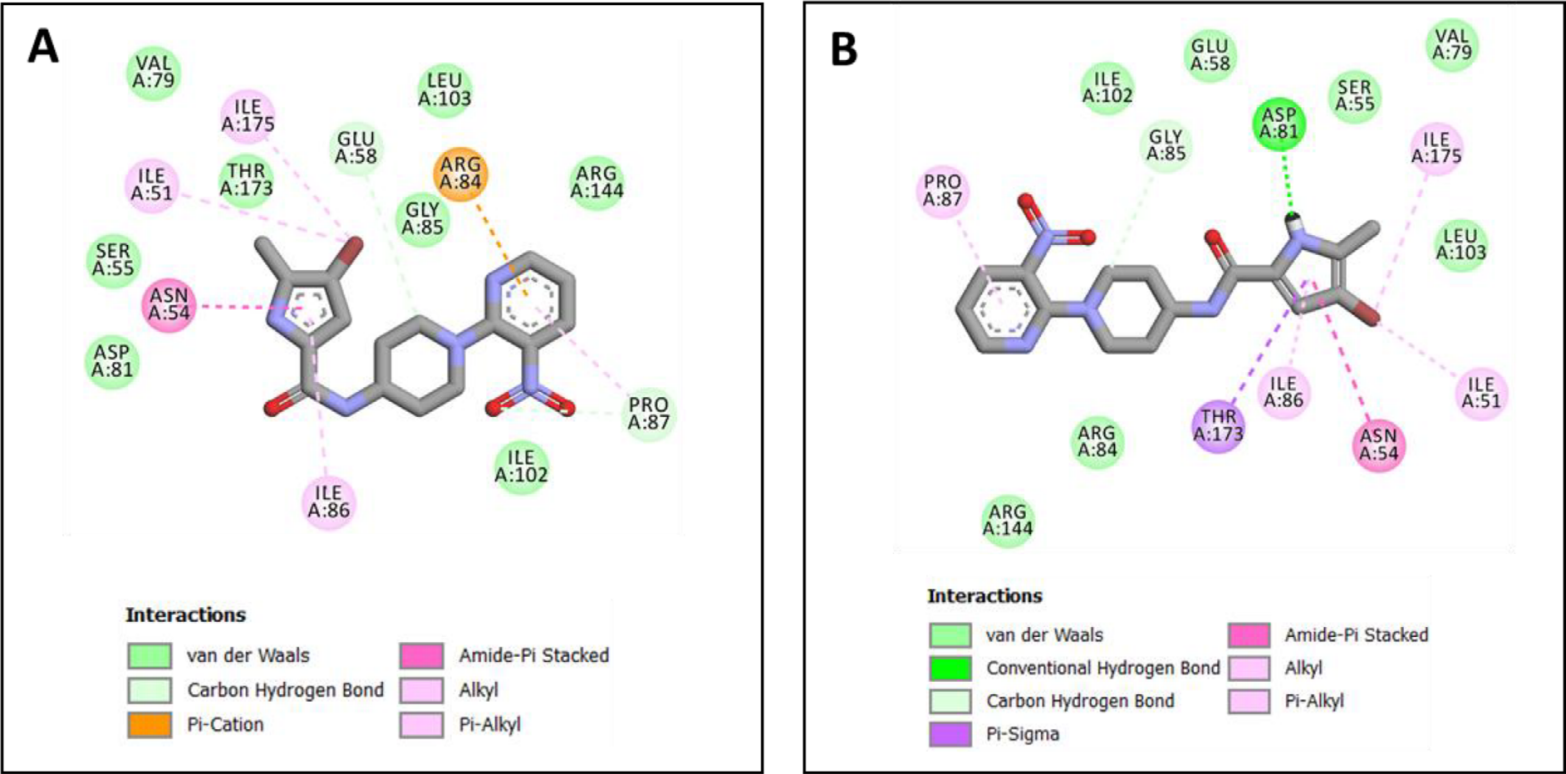
Binding modes of the **co‐crystallized ligand** (A) and **the redocked co‐crystallized ligand** (B) within the binding cavity of *Staphylococcus. aureus* GyrB ATPase.

**FIGURE 4:**
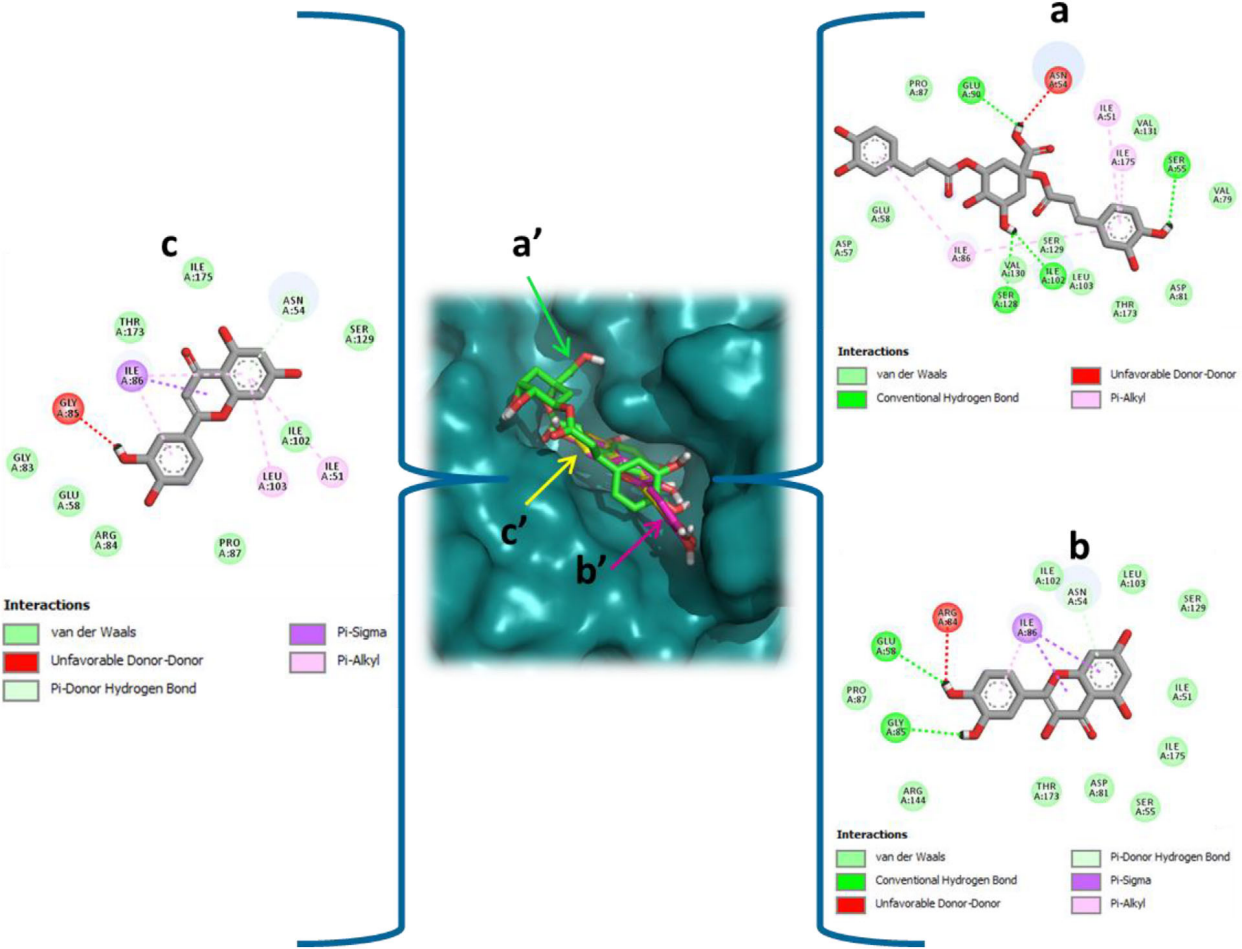
Binding modes of **1,3‐Di‐O‐caffeoquinic acid** (a), **Epicatechin** (b), **Quercetin‐ 3‐o‐rhamonoside** (c) and **Quercetin** (d) within the binding cavity of *Staphylococcus. aureus* GyrB ATPase.

**FIGURE 5:**
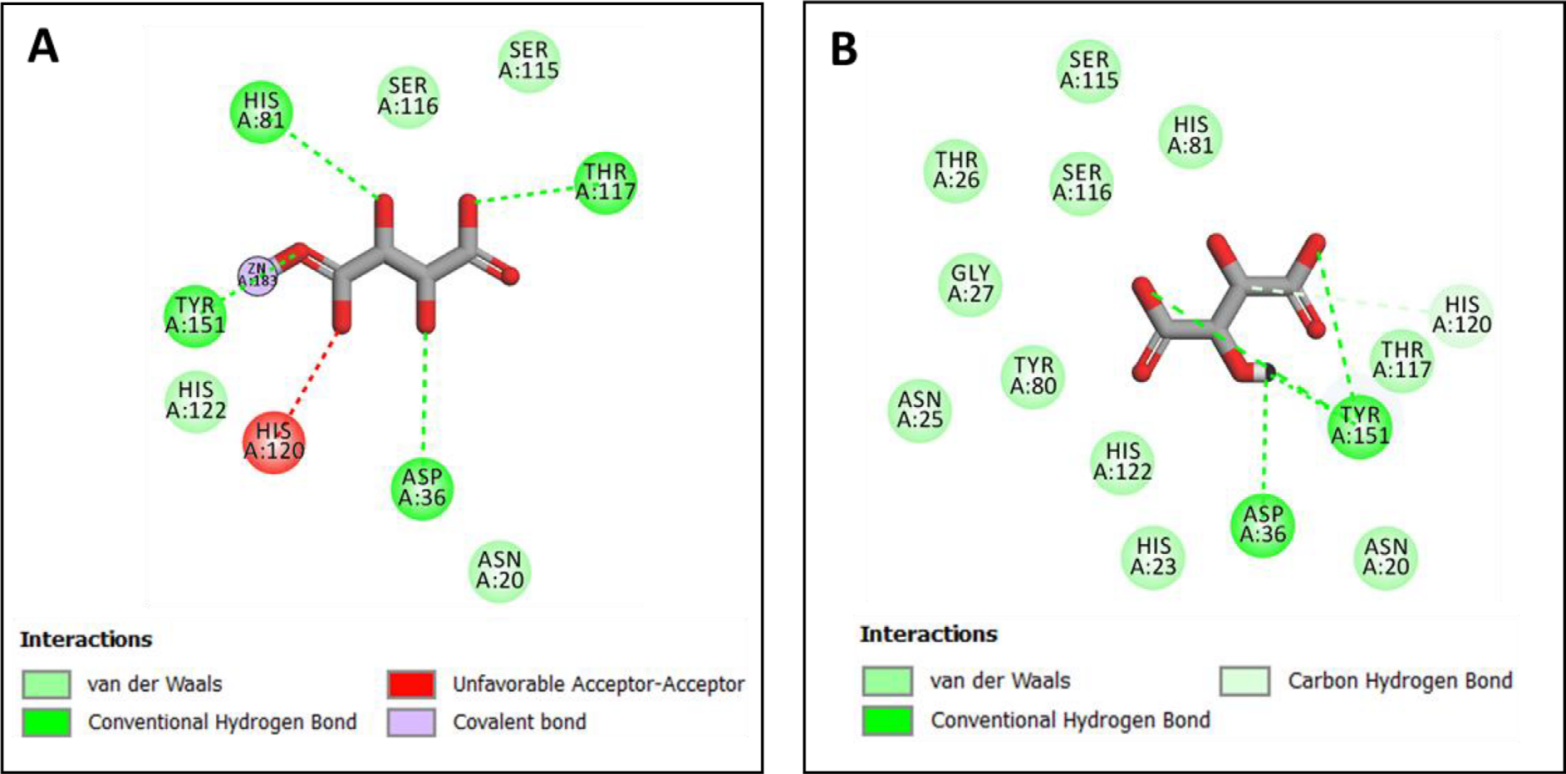
Binding modes of the **co‐crystallized ligand** (A) and **the redocked co‐crystallized ligand** (B) within the binding cavity of « LasA virulence factor from *Pseudomonas aeruginosa »*.

Protein‐ligand predicted docking score is very important for biological processes, because these physical and chemical interactions determine the biological recognition at the molecular level. Thus, in this way, it is possible to look for a ligand (tested compound) capable of inhibiting or activating a specific target protein via its interactions. By referring to bibliographic works, the docking study towards some target proteins of certain bacteria becomes an essential subject for evaluating the behavior of tested compounds to inhibit the bacterial effect of *S. aureus* [86], *P. aeruginosa* [87, 88], and *A. baumannii* [89, 90]. The crystal structures with PDB IDs 3U2D, 3IT7, and 3UDI, respectively were retrieved from the Protein Data Bank (PDB) for molecular docking studies. These structures were selected based on their high‐quality x‐ray crystallographic data, appropriate structural resolution, completeness of the binding site, and the availability of well‐characterized active‐site residues, making them suitable and reliable models for molecular docking simulations. To properly validate the molecular docking protocol, the binding mode of the co‐crystallized ligand was compared with that of the same ligand after redocking into the active site of the target protein. As illustrated in Figures 3, 5, and 7, the interaction profiles of the co‐crystallized ligand in the experimentally determined crystal structure and those of the redocked ligand are highly consistent. In both cases, the ligand maintains nearly identical orientations within the binding pocket and preserves the key molecular interactions with the surrounding amino acid residues, including hydrogen bonds, hydrophobic contacts, and other stabilizing interactions. This close agreement demonstrates that the docking protocol accurately reproduces the experimentally observed binding pose, thereby supporting the internal reproducibility of the docking protocol of the docking procedure for predicting ligand‐protein interactions and supporting its use in the subsequent virtual screening and binding affinity analyses. In such a way, it is essential to find a ligand that binds to the target protein with high affinity. All the above findings encouraged us to evaluate individually in silico the phytocompounds of the *C. verum* methanolic extract which is tested in vitro for its antibiofilm power. Indeed, against « *S. aureus* GyrB ATPase », quercetin was found to be the highest‐ranked ligand via the formation of two H‐bonds with Glu58 and Gly85 in addition to some other interactions as: Pi‐donor hydrogen bond (Asn54), Pi‐Sigma (Ile86), and Pi‐Alkyl (Ile86). Further, 1,3‐di‐O‐caffeoquinic acid and Luteolein were considered as the second compounds with the next most favorable predicted scores by showing some interesting interactions shown in Figure 4. Toward « LasA virulence factor from *Pseudomonas aeruginosa* » the docking outputs presented in Table 5 show that the best docking score was found with 1,3‐di‐O‐caffeoquinic acid (−8.4 kcal/mol). This ligand is involved in five H‐bonds with the amino acid sequence: Gln66, His81, Thr117, His120, and His122 besides to some other intermolecular contacts as shown inFigure 6 while the second next‐highest‐ranked compound quercetin‐ 3‐o‐rhamonoside (−8.1 kcal/mol) exhibited five hydrogen bonds with the residues: His23, Asp36, His120 and His122 in addition to Carbon hydrogen bond and Pi–Pi Stacked interactions with Tyr151. On another hand and as depicted in the tabulated results, against « *Acinetobacter baumannii* PBP1a », among all docked phytocompounds, the highest‐ranked compounds are 1,3‐di‐O‐caffeoquinic acid and quercetin‐ 3‐o‐rhamonoside by exhibiting the best docking score (binding energy value = −8.3 kcal/mol). The involved interactions depicted in Figure 8 demonstrate the formation of some interactions including several conventional hydrogen bonds and other stabilizing contacts, may account for the favorable predicted binding energy of the docking complex. By having a binding energy of −8.1 kcal/mol, epicatechin and quercetin were found to be the compounds with the second most favorable predicted scores. Therefore, these all above discussed molecules may have warrant subsequent biochemical and microbiological investigation especially by the interesting number of intermolecular hydrogen bonds formed.

**TABLE 5:** Binding energy values of the docked compounds.

Ligand	Binding energy (kcal/mol)		
	PDB : 3u2d	PDB : 3it7	PDB : 3udi
Quinic acid	−5.9	−6.2	−5.4
1,3‐di‐*O*‐caffeoquinic acid	−7.7	−8.4	−8.3
Gallic acid	−6.0	−5.9	−5.4
Protocachuic acid	−5.9	−5.4	−5.5
Chlorogenic acid	−7.4	−7.4	−7.6
Syringic acid	−5.5	−5.1	−5.1
Epicatechin	−7.5	−6.9	−8.1
*P*‐coumaric acid	−6.2	−5.3	−5.2
Trans‐frulic acid	−6.4	−5.4	−5.6
Rutin	−6.6	−7.7	−7.9
*O*‐coumaric acid	−6.1	−5.4	−5.4
quercetin‐ 3‐o‐rhamonoside	−7.4	−8.1	−8.3
Quercetin	−8.1	−7.1	−8.1
*Trans*‐cinnamic aldheyde	−5.1	−4.2	−5.4
Kaempferol	−7.6	−6.7	−7.7
Naringenin	−7.6	−6.8	−7.4
Luteolein	−7.7	−7.2	−7.8
Co‐crystallized ligand	−6.9	−4.6	−6.6

**FIGURE 6:**
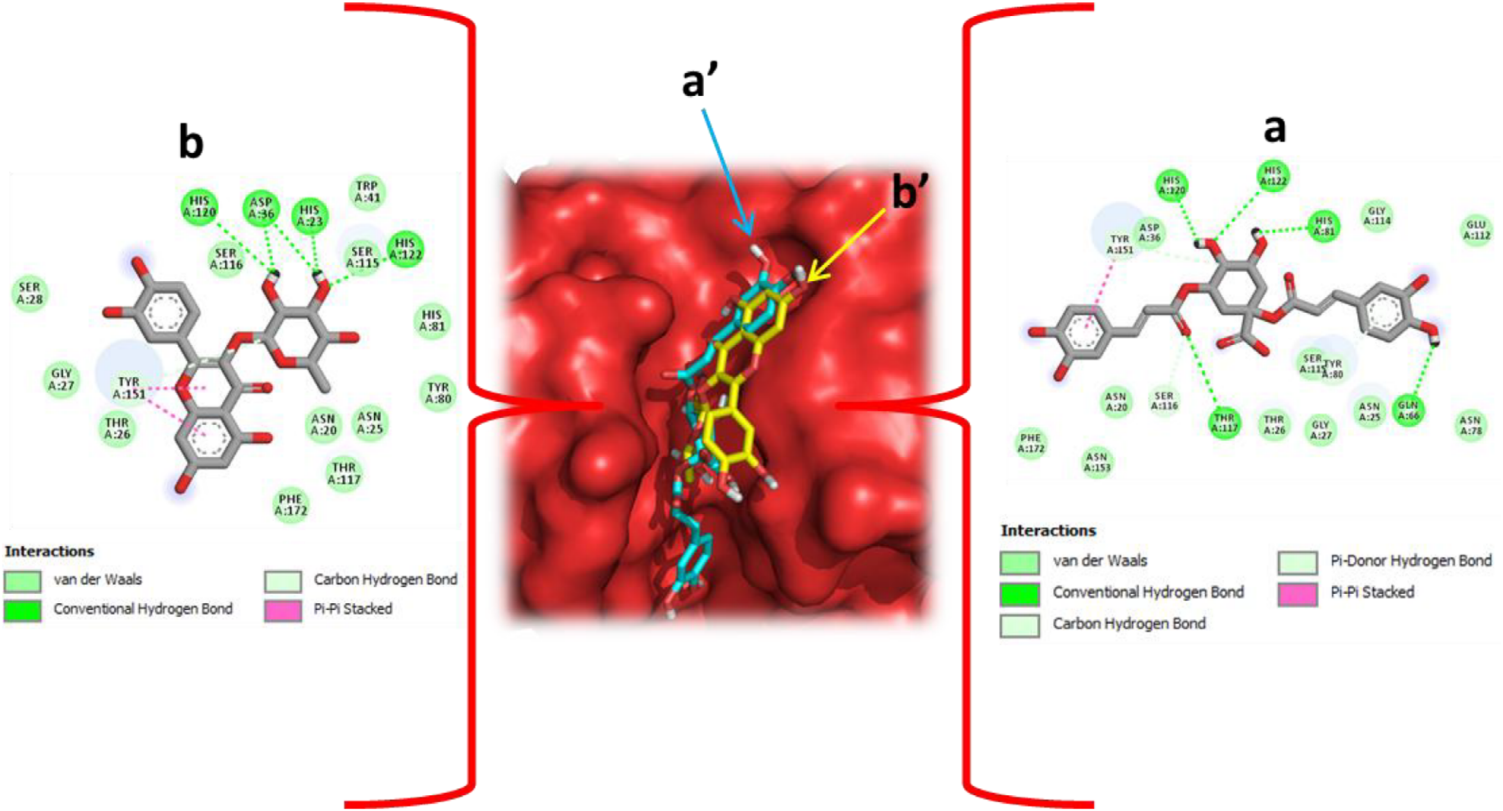
Binding modes of 1,3‐di‐O‐caffeoylquinic acid (a) and quercetin‐3‐O‐rhamnoside (b) within the binding cavity of the LasA virulence factor from *Pseudomonas aeruginosa*.

**FIGURE 7:**
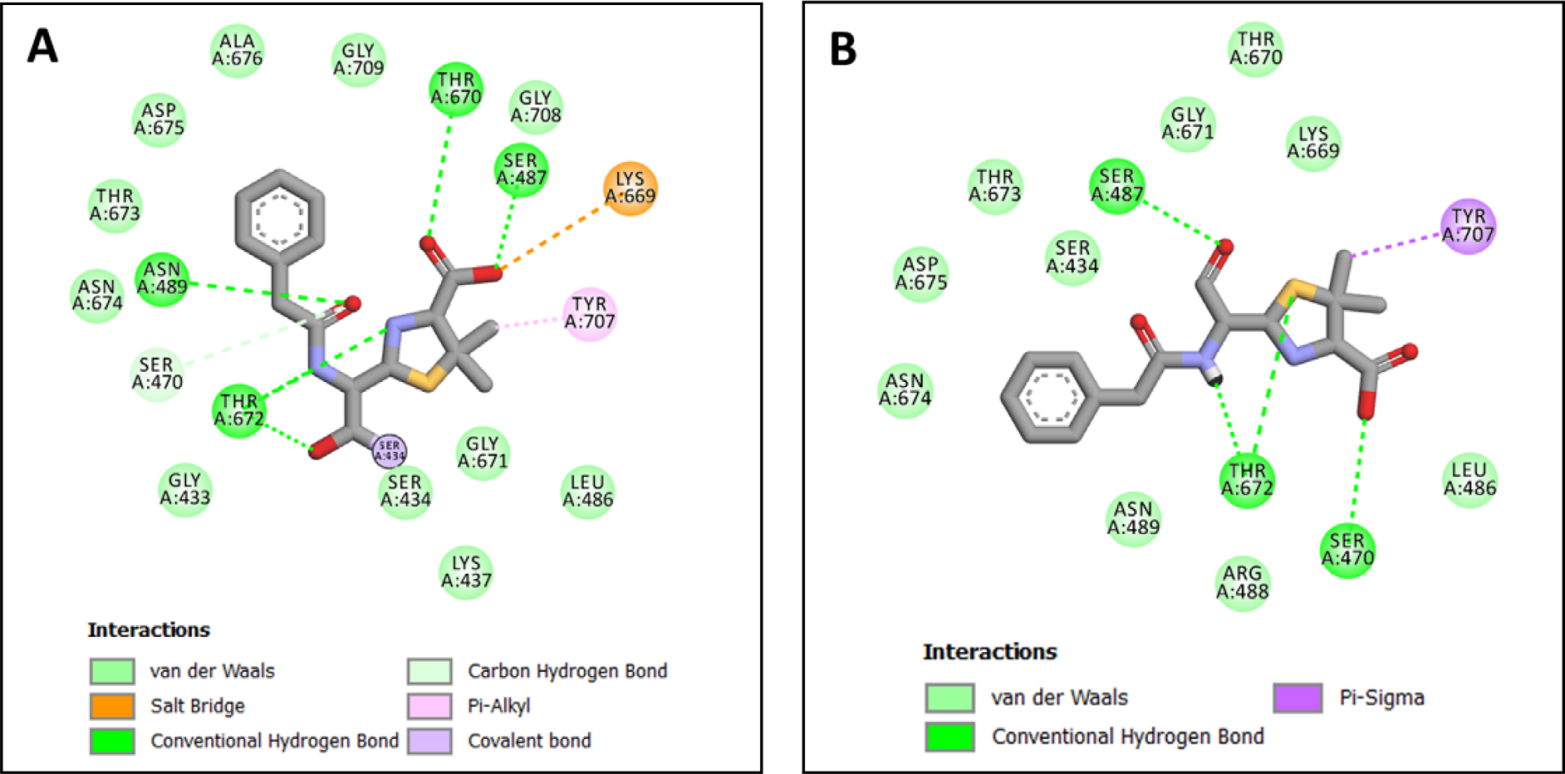
Binding modes of the co‐crystallized ligand (A) and the redocked co‐crystallized ligand (B) within the binding cavity of *Acinetobacter baumannii* PBP1a.

**FIGURE 8:**
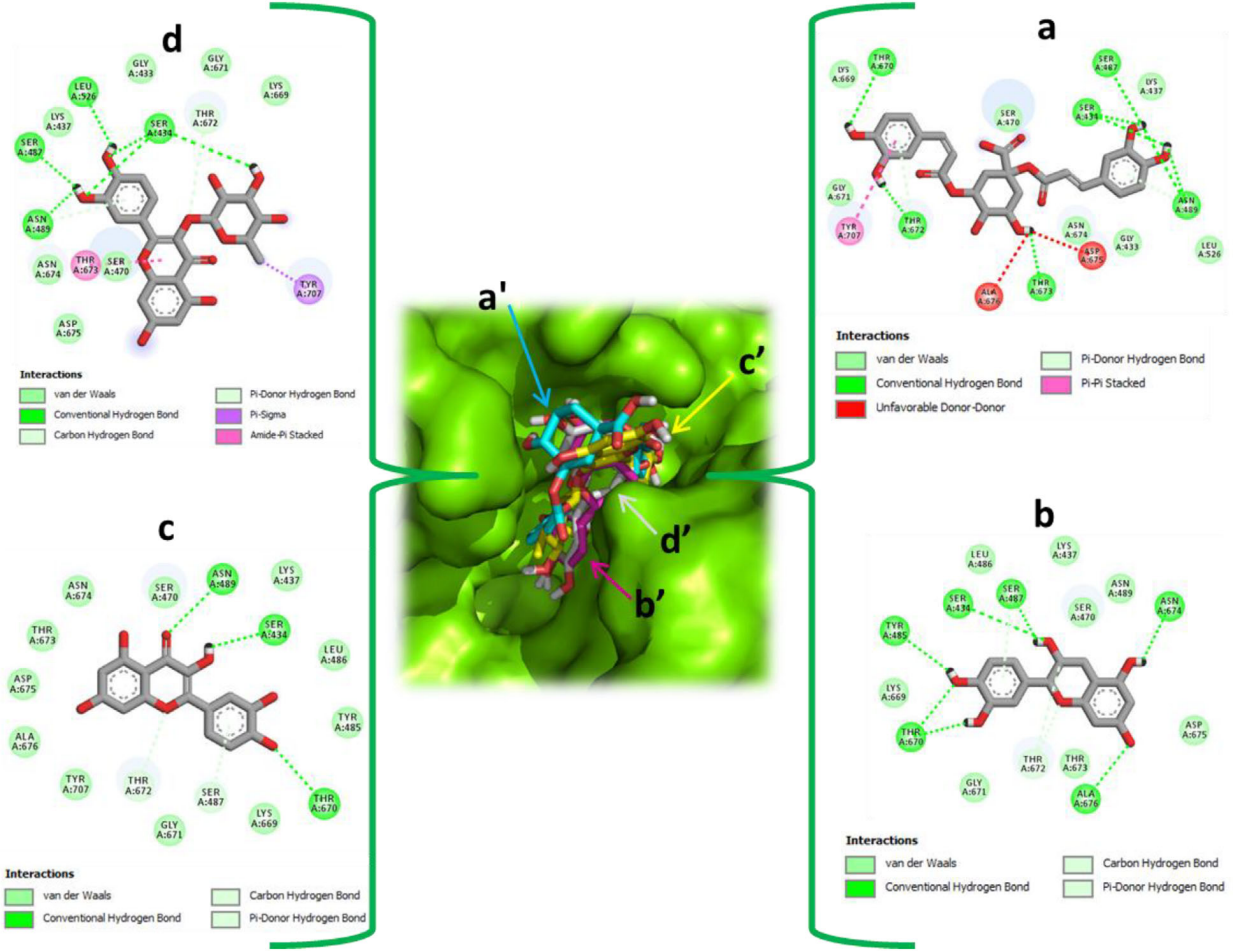
Binding modes of 1,3‐di‐O‐caffeoylquinic acid (a), quercetin‐3‐O‐rhamnoside (b), epicatechin (c), and quercetin (d) within the binding cavity of *Acinetobacter baumannii* PBP1a.

## 4 Conclusion

The methanolic bark extract of *Cinnamomum verum* contained 17 identified constituents was dominated by *trans*‐cinnamaldehyde, and exerted bactericidal activity against reference and multidrug‐resistant bacteria. The particularly low MIC values recorded for imipenem‐resistant *Acinetobacter baumannii* and the concentration‐dependent reduction in biofilm biomass demonstrate that the extract affected multiple antibacterial phenotypes, although these responses remained strain dependent. Disk‐diffusion screening identified promising antibiotic‐extract combinations, including cefoxitin and fosfomycin against MRSA and selected combinations against *A. baumannii*, but also revealed unchanged or reduced inhibition zones for other combinations. Therefore, the extract should not be considered a universal antibiotic enhancer, and the observed increases represent preliminary potentiation rather than confirmed synergy. The docking results further prioritized quercetin, 1,3‐di‐O‐caffeoylquinic acid, and quercetin‐3‐O‐rhamnoside for mechanistic investigation, without constituting evidence of target inhibition. The principal advance of this work is the integration, within the same chemically characterized extract, of phytochemical profiling, MIC/MBC determination, biofilm‐biomass assessment, antibiotic‐interaction screening, and exploratory target‐based modeling across bacterial species with distinct resistance phenotypes. This comparative framework expands the available evidence for *C. verum* against clinically relevant resistant bacteria, particularly *A. baumannii*, and provides a focused shortlist of extract constituents and antibiotic combinations for subsequent validation. Nevertheless, the mg/mL active concentrations and the absence of checkerboard, time‐kill, cytotoxicity, selectivity‐index, and experimental target‐inhibition data currently preclude therapeutic extrapolation. Fractionation, compound‐combination studies, mechanistic assays, and in vivo validation are required to determine whether these findings can be translated into safe and effective antimicrobial‐adjuvant strategies.

## Author Contributions

H.E., S.E., A.K., M.H., H.N., N.N., H.A., M.N.A., R.A., G.F., I.O.A., and M.S.O. contributed to the conception and design of the study, experimental work, data analysis and interpretation, and manuscript preparation. H.E., I.O.A., and M.S.O. coordinated and supervised the study. All authors discussed the results, critically revised the manuscript, and read and approved the final version.

## Funding

This research received no external funding.

## Conflicts of Interest

The authors declare no conflicts of interest.

## Data Availability

The data supporting the findings of this study are available from the corresponding author upon reasonable request.
